# Population pharmacokinetic and exploratory exposure–response analysis of the fixed-dose combination of pertuzumab and trastuzumab for subcutaneous injection in patients with HER2-positive early breast cancer in the FeDeriCa study

**DOI:** 10.1007/s00280-021-04296-0

**Published:** 2021-06-09

**Authors:** Bei Wang, Rong Deng, Stefanie Hennig, Tanja Badovinac Crnjevic, Monika Kaewphluk, Matts Kågedal, Angelica L. Quartino, Sandhya Girish, Chunze Li, Whitney P. Kirschbrown

**Affiliations:** 1 Genentech, Inc., 1 DNA Way, South San Francisco, CA 94080 USA; 2 Certara, Inc., Princeton, NJ USA; 3 F. Hoffmann-La Roche Ltd, Basel, Switzerland; 4 Present Address: Clinical Pharmacology and Quantitative Pharmacology, AstraZeneca, Gothenburg, Sweden; 5 Present Address: Gilead Sciences, Inc., Foster City, CA USA

**Keywords:** Exposure–response analysis, Fixed-dose combination, HER2-positive, Pertuzumab, trastuzumab, and hyaluronidase-zzxf, Population pharmacokinetic model, Subcutaneous

## Abstract

**Purpose:**

To characterize pertuzumab pharmacokinetics (PK) in FeDeriCa (NCT03493854: fixed-dose combination of pertuzumab and trastuzumab for subcutaneous injection [PH FDC SC] versus intravenous pertuzumab plus trastuzumab); derive individual pertuzumab exposures in the PH FDC SC arm for subsequent pertuzumab exposure–response (ER) analyses; compare observed trastuzumab PK with predicted exposures from a previous SC trastuzumab model; assess whether pertuzumab affects trastuzumab PK; evaluate pertuzumab exposure–efficacy and –safety relationships and support the approved SC dosing regimen.

**Methods:**

Population pharmacokinetic modeling and simulations were used to describe the data. Standard goodness-of-fit diagnostics and prediction-corrected visual predictive checks were used for model performance assessment. Covariates were included from previously reported models. ER analysis was conducted using logistic regression.

**Results:**

SC pertuzumab PK was described adequately by a two-compartment model with first-order absorption; significant covariates included in the final model were albumin, lean body weight, and Asian region; however, these appeared not to be clinically relevant. Trastuzumab concentrations were described adequately by the previous model; there was no evidence of a pertuzumab effect on trastuzumab PK as part of PH FDC SC and higher model-predicted pertuzumab exposure was not associated with differences in pathologic complete response rate or an increased probability of selected grade ≥ 3 adverse events of interest.

**Conclusion:**

The approved PH FDC SC dose [loading: 1200/600 mg pertuzumab/trastuzumab (15 mL); maintenance: 600 mg pertuzumab/trastuzumab (10 mL) and 2000 U/mL recombinant human hyaluronidase every 3 weeks] provides a positive benefit–risk profile with comparable efficacy and safety to intravenous pertuzumab plus trastuzumab.

**Supplementary Information:**

The online version contains supplementary material available at 10.1007/s00280-021-04296-0.

## Introduction

Pertuzumab (PERJETA^®^, F. Hoffmann-La Roche Ltd, Basel, Switzerland) and trastuzumab (Herceptin^®^, F. Hoffmann-La Roche Ltd) have complementary mechanisms of action [1, 2], with in vivo studies showing that they bind to different epitopes on the human epidermal growth factor receptor 2 (HER2) protein. Whereas trastuzumab binds to the extracellular domain of HER2 and inhibits ligand-independent signaling, pertuzumab prevents dimerization of HER2 with other family proteins and, thus, downstream signaling processes associated with tumor growth and progression [3–5]. Accordingly, adding intravenous pertuzumab to intravenous trastuzumab (P + H IV) and chemotherapy significantly improves efficacy compared with trastuzumab and chemotherapy in first-line metastatic breast cancer, and in the neoadjuvant and adjuvant early breast cancer settings [6–13].

A fixed-dose combination of pertuzumab and trastuzumab for subcutaneous injection [pertuzumab, trastuzumab, and hyaluronidase-zzxf (PHESGO™, F. Hoffmann-La Roche Ltd; PH FDC SC)] has been recently approved by several health authorities, including the U.S. Food and Drug Administration (FDA) and the European Medicines Agency. PH FDC SC for the first time combines two monoclonal antibodies in one vial and contains pertuzumab, trastuzumab, and recombinant human hyaluronidase (rHuPH20; an enzyme that temporarily degrades hyaluronan under the skin to aid in the dispersion and absorption of the antibodies) in a ready-to-use formulation. PH FDC SC demonstrates clear time-saving advantages compared with P + H IV, as well as other advantages over IV infusion such as less invasiveness, reduced pain, and improved convenience through at-home administration options (via a visiting healthcare professional) [14–16]. The approved SC formulation of trastuzumab has also been shown to have non-inferior serum trough concentrations (C_trough_) and pathologic complete response (pCR) rates to IV trastuzumab, along with consistent long-term efficacy and a comparable safety profile [17–26]. The pharmacokinetics (PK) of IV pertuzumab, SC pertuzumab, and SC trastuzumab have been studied extensively [27–29] and support their comparable efficacy and safety profiles.

The pertuzumab and trastuzumab doses in the PH FDC SC were supported by population PK (popPK) modeling and simulation data in solid tumors: IV pertuzumab PK was described by a two-compartment linear model with first-order elimination, with baseline serum albumin and lean body weight (LBW) having statistically significant, but not clinically relevant, effects on IV pertuzumab clearance [27]. A phase Ib study in healthy male volunteers and female patients with breast cancer showed that an SC pertuzumab 600 mg maintenance dose had equivalent exposure [C_trough_ and area under the curve (AUC)] to IV pertuzumab at 420 mg with no new safety signals [28]. The SC trastuzumab dose within PH FDC SC was based on an analysis from the HannaH study, where a 600 mg fixed dose was confirmed to give the desired exposure [29]. As such, the PH FDC SC is given as a loading dose of 1200 mg pertuzumab and 600 mg trastuzumab in 15 mL, and 600 mg pertuzumab and trastuzumab maintenance doses in 10 mL, along with 2000 U/mL rHuPH20 every 3 weeks. This dose was assessed clinically in the FeDeriCa study (NCT03493854), which showed that PH FDC SC was non-inferior to P + H IV, based on cycle 7 pertuzumab serum C_trough_ concentrations, and that PH FDC SC and P + H IV demonstrated comparable efficacy (total pathologic complete response) and safety profiles [30].

Here, we present three analyses of the FeDeriCa study. First, a popPK analysis aiming to characterize the PK of pertuzumab after P + H IV and PH FDC SC administration and to derive individual pertuzumab exposures in the PH FDC SC arm for subsequent exposure–response (ER) analyses. Second, a comparison of the observed PK of trastuzumab in the PH FDC SC arm with predicted trastuzumab exposures based on a previously established popPK model of SC trastuzumab, to assess whether pertuzumab affects trastuzumab PK in the PH FDC SC arm [29]. Third, a pertuzumab ER analysis in the PH FDC SC arm, which aimed to evaluate the pertuzumab exposure–efficacy and –safety relationships and, thus, confirm the dosing regimen and support the label for this new formulation.

## Materials and methods

FeDeriCa was conducted in conformance with Good Clinical Practice guidelines and the Declaration of Helsinki [30]. The protocol was reviewed and approved by the institutional review board or ethics committee at each study site; all patients provided written informed consent [30].

### Study design [30]

FeDeriCa is a randomized, open-label, international, multicenter, two-arm phase III non-inferiority trial of PH FDC SC compared to P + H IV in the neoadjuvant–adjuvant early breast cancer setting. Patients were randomized 1:1 to P + H IV or PH FDC SC, with stratification based on hormone receptor status (estrogen receptor- or progesterone receptor-positive, or estrogen receptor- and progesterone receptor-negative), clinical stage (II–IIIA or IIIB–IIIC), and chemotherapy regimen [4 cycles of dose-dense doxorubicin plus cyclophosphamide (ddAC) every 2 weeks followed by paclitaxel once weekly for 12 weeks, or 4 cycles of doxorubicin plus cyclophosphamide (AC) every 3 weeks followed by 4 cycles of docetaxel every 3 weeks].

### Patients [30]

Patients had HER2-positive (immunohistochemistry 3+ or in situ hybridization-positive; centrally confirmed along with hormone receptor status) early breast cancer, were candidates for preoperative neoadjuvant treatment, aged ≥ 18 years, and had operable, locally advanced, or inflammatory stage II–IIIC disease with a primary tumor > 2 cm in diameter, or node-positive disease. Patients also had an Eastern Cooperative Oncology Group (ECOG) performance status of 0 or 1 and a left ventricular ejection fraction of ≥ 55% (by echocardiography or multiple-gated acquisition scan). Exclusion criteria included receipt of any systemic therapy for treatment or prevention of breast cancer, or radiation treatment for cancer, serious cardiac conditions, impaired liver function, or inadequate renal or bone marrow function.

### Procedures [30]

Investigators chose one of two chemotherapy regimens before randomization: 4 cycles of ddAC comprising 60 mg/m^2^ doxorubicin plus 600 mg/m^2^ cyclophosphamide every 2 weeks (with local guideline-specified granulocyte colony-stimulating factor if needed, per local guidelines) followed by 80 mg/m^2^ paclitaxel once a week for 12 weeks, or 4 cycles of AC comprising 60 mg/m^2^ doxorubicin plus 600 mg/m^2^ cyclophosphamide every 3 weeks followed by 4 cycles of 75–100 mg/m^2^ docetaxel every 3 weeks. Following anthracycline-based chemotherapy, and concomitant with the taxane chemotherapy, patients received four cycles of HER2-targeted therapy (given before the taxane on the same day). P IV was given as an 840 mg loading dose followed by 420 mg maintenance doses every 3 weeks; H IV, as an 8 mg/kg loading dose followed by 6 mg/kg maintenance doses every 3 weeks (the order being according to investigator preference). PH FDC SC was given as a 1200 mg P/600 mg H loading dose in 15 mL followed by 600 mg P/600 mg H maintenance doses in 10 mL every 3 weeks. After completion of this neoadjuvant component of the study, patients received surgery, followed by 14 three-weekly HER2-targeted therapy cycles as randomized to complete a maximum total of 18 cycles. Treatment was discontinued for instances of investigator-assessed radiographic or clinical disease progression or recurrence or unmanageable toxicity. Dose modifications of PH FDC SC, P IV, or H IV were not allowed. Patients were withdrawn from all study treatment if the HER2-targeted component was withheld for more than 2 cycles (i.e., more than 9 weeks between doses) or if HER2-targeted therapy needed to be permanently discontinued for treatment-related toxicity.

This analysis is based on the neoadjuvant period only. During this period, blood samples for PK assessments were collected pre- and post-dose on day 1 in the P + H IV arm in cycles 5, 6, 7, 8, and on days 2, 4, 8, and 15 of cycle 7. In the PH FDC SC arm, samples were collected pre-dose on day 1 of cycles 5, 6, 7, and 8, on days 2 and 15 of cycle 5, and on days 2, 4, 8, and 15 of cycle 7. Actual collection times were recorded. A validated duplex hybrid immunoaffinity capture liquid chromatography tandem mass spectrometry assay was used to simultaneously measure the concentration of both pertuzumab and trastuzumab in serum samples. The concentration of the lower limit of quantification (LLQ) for both analytes was 100 ng/mL.

### Pertuzumab population pharmacokinetic analysis

PK analyses included both SC and IV data and patients. Patients were included in the popPK analysis if they had at least one PH FDC SC or P + H IV administration and a corresponding PK sample collection after dosing with a concentration above the LLQ. Samples were included from cycle 5 through to day 1 of cycle 8. If the time of either drug administration or sample collection was missing, the record was excluded from analysis. Patients who had at least one observed post-dose pertuzumab concentration and are included as part of the final popPK model were included in the ER analysis (patients in the PH FDC SC arm only). Any observations below the LLQ, of which there were 16 (0.31%), were omitted across datasets.

A two-compartment model with first-order absorption for SC administration and first-order elimination from the central compartment was utilized as a starting point for the modeling using NONMEM version 7.4.3 (ICON Development Solutions, Ellicott City, Maryland, USA) with the first-order conditional estimation with interaction method. Evaluated covariates included those from previous pertuzumab IV models (LBW, albumin), standard demographic variables (age, sex, race), standard baseline laboratory variables (aspartate aminotransferase, alanine aminotransferase, total protein, total bilirubin, alkaline phosphatase, creatinine, creatinine clearance), stratification factors in the study design [hormone receptor status (estrogen receptor- or progesterone receptor-positive, or estrogen receptor- and progesterone receptor-negative), clinical stage (II–IIIA or IIIB–IIIC), and chemotherapy regimen (4 cycles of ddAC every 2 weeks followed by paclitaxel once weekly for 12 weeks, or 4 cycles of AC every 3 weeks followed by 4 cycles of docetaxel every 3 weeks)], standard disease-related variables (ECOG), or covariates of specific interest (Asian region). Covariates were selected using a forward addition and backward elimination stepwise procedure (significance levels set to *p* < 0.01 and *p* < 0.001, respectively). Potential outliers were excluded based on conditional-weighted residuals (|CWRES |> 5) after the initial base model assessment. Models were evaluated using standard goodness-of-fit diagnostics and prediction-corrected visual predictive checks (pcVPC). Model applications included a display of the magnitude of covariate effects using forest plots, and exposure-comparisons using post hoc exposure estimates stratified by covariates of interest.

### Trastuzumab population pharmacokinetic simulations for assessment of drug–drug interactions

The H PK dataset included patients from the FeDeriCa study who had at least one administration of PH FDC SC and one post-dose trastuzumab PK sample above the LLQ. Samples were included from cycle 5 through to day 1 of cycle 8. The HannaH popPK model was a two-compartment model with parallel linear and nonlinear elimination, and first-order absorption for SC administration [29]. This model was used to simulate trastuzumab exposures based on the FeDeriCa study design and patient characteristics (dosing regimens, baseline covariates, and sample times). Trastuzumab concentration predictions from the HannaH PK model and the observed trastuzumab concentrations from FeDeriCa were then compared visually via a pcVPC and numerically using a numerical predictive check (NPC). This was done using NONMEM version 7.4.3 and Perl Speaks NONMEM version 4.8.1 (Uppsala University, Sweden).

### Exposure–response and exposure–safety analyses

The datasets included all patients who received PH FDC SC and had one post-dose pertuzumab concentration above the LLQ. The post hoc PK parameter estimates from these patients were used to generate popPK model-predicted exposures. Exposure metrics included model-predicted cycle 7 C_trough_ (pre-dose cycle 8) for efficacy analyses, cycle 7 maximum concentration (C_max_) for safety analyses, and cycle 7 AUC for both. Cycle 7 represents three doses of pertuzumab (1200 mg loading dose at cycle 5 and 600 mg maintenance dose every 3 weeks thereafter), and based on PK simulation, the majority of patients reached C_trough_ steady state at cycle 7. Exploratory analysis included exposure boxplots versus event status and event rates versus mean exposure stratified by exposure quartiles. Modeling was conducted using logistic regression (R version 3.4.3). If an ER relationship was observed, covariate testing was performed using a forward–backward stepwise search method (significance levels of *p* < 0.05 and *p* < 0.01, respectively). Covariates included standard demographic variables (age, sex, race), ECOG performance status, and study stratification variables. Response rates were projected for populations and in different exposure quartiles relative to the overall population, for endpoints with a significant exposure–efficacy relationship. Safety events used in this analysis included grade ≥ 3 adverse events (AEs), serious AEs, grade ≥ 3 diarrhea, grade ≥ 3 neutropenia, cardiac toxicity, injection-related reactions, and hypersensitivity; all of which occurred between cycle 5 and cycle 7 C_trough_ measurement.

## Results

### Patients and samples

Five hundred patients were randomized [30], and 5656 pertuzumab serum PK samples were collected from 489 patients. A summary of baseline demographics and clinical characteristics is shown in Online Resource 1. Nine patients were not sampled, one had no post-dose sample above the LLQ, and one did not provide PK samples in cycles 5 to 8. Of the 489 patients with pertuzumab PK samples, 246 (50.3%) were randomized to the P + H IV arm and 243 (49.7%) to the PH FDC SC arm. After excluding pre-dose pertuzumab serum PK samples prior to the first dose in cycle 5 (*n* = 460), a further 16 (0.31%) samples below the limit of quantification (seven in the PH FDC SC arm) were identified and removed. This resulted in a total of 5180 evaluable pertuzumab serum PK samples for the analysis, of which 2093 observations were after SC injection and 3087 after IV infusion.

### Pertuzumab population pharmacokinetic analysis

Overall, 40/5180 (0.8%) PK samples were excluded from the analysis based on conditional-weighted residuals (|CWRES|> 5) after the initial base model assessment (an initial 2-compartment model with linear elimination from the central compartment, first-order absorption of the SC formulation, and a proportional residual error model); among them, there were 7 (0.14%) outliers after SC administration and 33 (0.64%) outliers after IV administration. The final model building dataset contained 489 patients and 5140 pertuzumab serum PK samples. In the forward stepwise covariate modeling step, in addition to LBW on clearance (CL), central volume (*V*_*c*_), and peripheral volume (*V*_*p*_) and albumin on CL, Asian (region) was added as significant covariate (*p* < 0.01) on CL. No covariate was excluded through the backward step. Final model parameters are summarized in Table 1. Covariate relationships and parameter values are described by the equations below; the final bioavailability (*F*) was 0.712:

$${\text{CL }}\left( {\text{L/days}} \right) =\, 0.163 \times \left( {{\text{ALB}}/43.25} \right)^{ - 0.629} \times \left( {{\text{LBW}}/45.09} \right)^{1.252} \times \left( {1.123\, {\text{if in Asian region}}} \right) \,\, \times e^{\eta CL} ,$$

$$V_{c} \,\left( {\text{L}} \right) = 2.77 \times \left( {{\text{LBW}}/45.09} \right)^{0.839} \times e^{\eta V2} ,$$

$$Q\, \left( {\text{L/days}} \right) = 0.616,$$

$$V_{p}\, \left( {\text{L}} \right) = 2.49 \times \left( {{\text{LBW}}/45.09} \right)^{0.716} \times e^{\eta V3} ,$$

$$F = 0.712 \times e^{\eta F} .$$

**Table 1:** Final population pharmacokinetic model parameter estimates

Parameter	Parameter description	Estimate (95% CI)	RSE for estimate, %	IIV, CV% (95% CI)	RSE for IIV, %	Shrinkage, %
θ_1_	CL, L/d	0.163 (0.144, 0.182)	5.81	23.5 (4.89, 32.8)	48.8	14.4
θ_2_	*V*_*c*_, L	2.77 (2.60, 2.94)	3.15	34.8 (32.1, 37.3)	7.52	11.2
θ_3_	*Q*, L/d	0.616 (0.553, 0.679)	5.23	–	–	–
θ_4_	*V*_*p*_, L	2.49 (2.11, 2.87)	7.69	25.6 (23.5, 27.5)	7.93	49.7
θ_5_	*k*_a_, d	0.348 (0.295, 0.401)	7.72	–	–	–
θ_6_	*F*	0.712 (0.489, 0.935)	16.0	17.8 (17.6, 18.0)	1.10	50.2
Residual error						
θ_7_	Proportional residual error (SC)	0.155 (0.142, 0.168)	4.32	–	–	10.1
θ_8_	Proportional residual error (IV)	0.175 (0.160, 0.190)	4.41	–	–	10.1
Covariates						
θ_9_	Albumin on CL	–0.629 (–0.221, –1.04)	33.1	–	–	–
θ_10_	LBW on CL	1.25 (0.922, 1.58)	13.4	–	–	–
θ_11_	LBW on *V*_*c*_	0.839 (0.545, 1.13)	17.9	–	–	–
θ_12_	LBW on *V*_*p*_	0.716 (0.251, 1.18)	33.1	–	–	–
θ_13_	Change of CL in the Asian region	0.123 (0.049, 0.197)	31.0	–	–	–
Half-life, d		24.3	–	28.2	–	–

The half-life estimate was calculated from typical parameter values. The half-life IIV was calculated from post hoc estimates

*CL* is clearance, *CV* is coefficient of variation, *d* is days, *F* is bioavailability, *IIV* is inter-individual variability, *IV* is intravenous, *LBW* is lean body weight, *k*a is apparent first-order absorption, *Q* is inter-compartmental clearance, *RSE* is relative squared error, *SC* is subcutaneous, *V*_*c*_ is central volume, *V*_*p*_ is peripheral volume

Parameters were well estimated with all relative standard errors < 35% for fixed-effects parameters (Table 1).

**Fig. 1:**
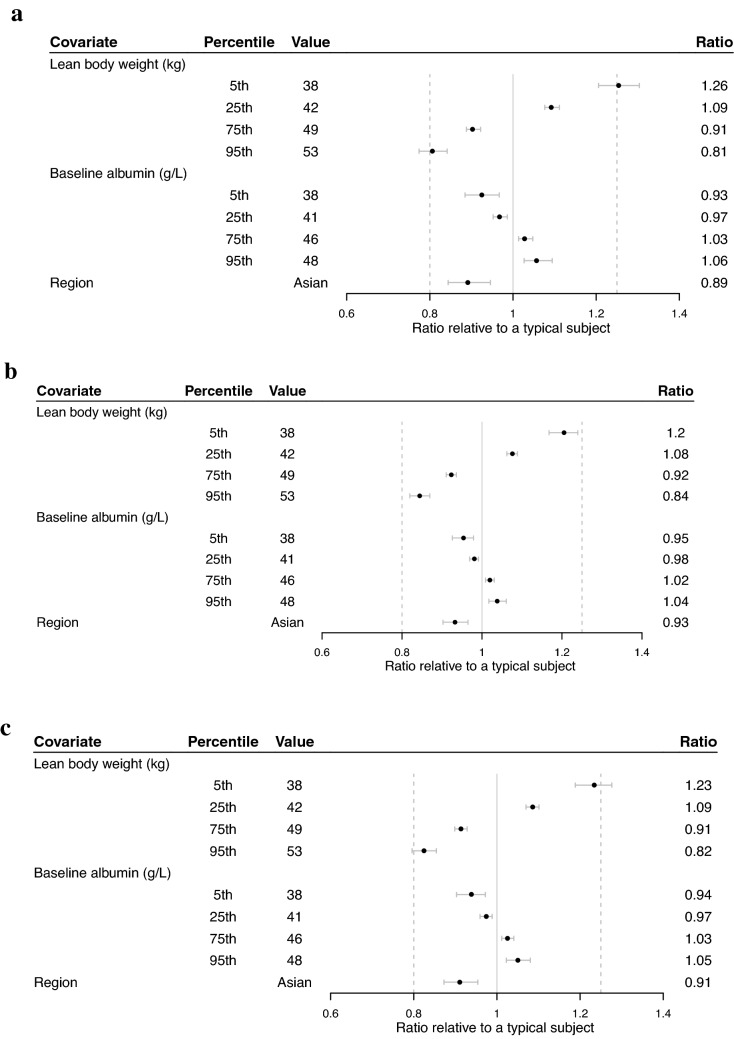
Effects of covariates on pertuzumab exposure metrics [C_trough_ (**A**), C_max_ (**B**), and AUC (**C**)] at cycle 7. Points represent the estimated exposure ratio for the patient with indicated covariate value relative to a typical patient with lean body weight 45.1 kg, albumin 43.3 g/L, who is non-Asian, receiving a dose of pertuzumab at 1200 mg in cycle 5 and 600 mg in cycles 6 and 7 as part of their PH FDC SC administration. The horizontal bars represent 90% confidence intervals. The vertical dashed lines at 0.8 and 1.25 indicate standard bioequivalence ratio bounds. The vertical line at one indicates a null effect. *AUC* is area under the curve for a dosing interval (tau = 21). *C*_*max*_ is maximum concentration. *C*_*trough*_ is trough concentration. Created using R version 3.6.0 (2019-04-26) in RStudio version 1.2.1335

CL decreased in patients with higher albumin concentrations and increased in patients with greater LBW. Both *V*_*c*_ and *V*_*p*_ increased in patients with greater LBW. CL was approximately 10% greater in patients in the Asian region. All exposure ratios fell within the 0.8–1.25 boundary relative to a typical patient, except for a patient at the 5th percentile LBW of 38 kg relative to a typical patient with LBW 45.1 kg (ratio of 1.26) (Fig. 1).

**Fig. 2:**
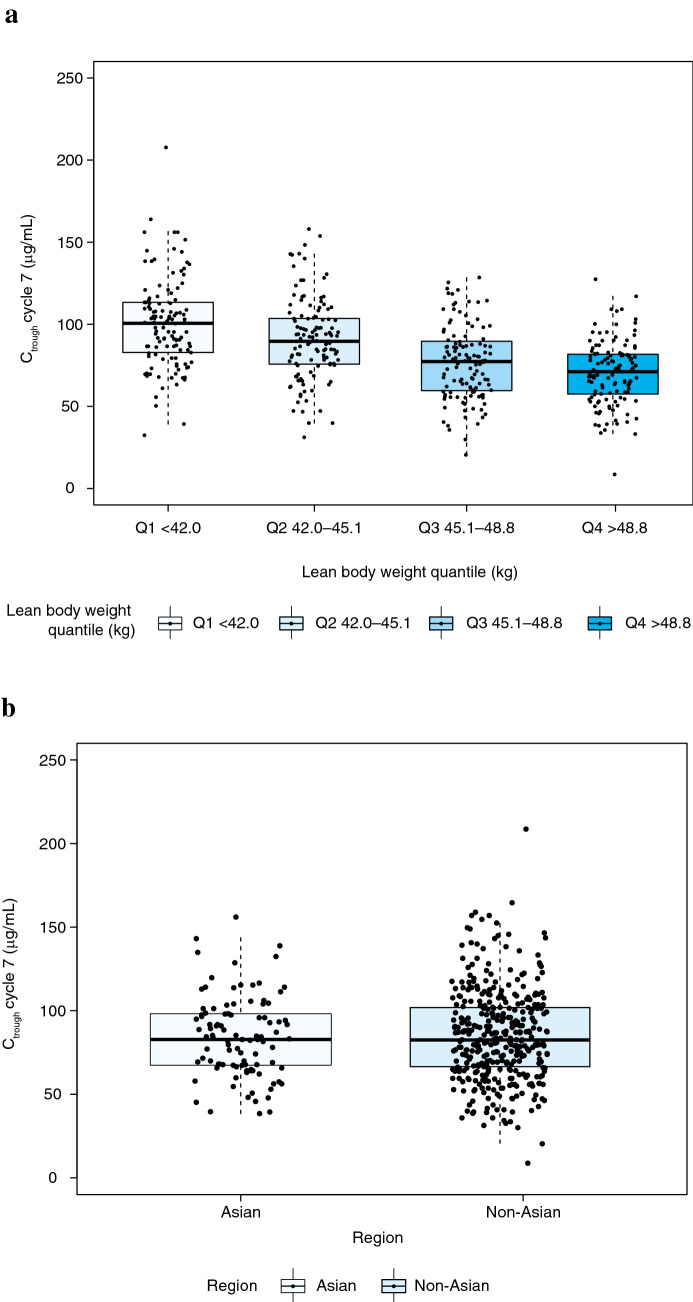
Model-predicted cycle 7 C_trough_ by lean body weight quartile (**A**) and Asian region (**B**). Solid bold lines represent medians. Shaded boxes represent the interquartile ranges. Whiskers represent 1.5 times the interquartile range. Points are jittered in the x-axis direction for clarity. C_trough_ were generated using individual empirical Bayes estimates. *C*_*trough*_ is trough concentration. Created using R version 3.6.0 (2019-04-26) in RStudio version 1.2.1335

As expected, heavier patients had lower exposures as described by the LBW effect on CL (Fig. 2). Although CL was greater in the Asian region, patients in that region had similar trough levels to patients in the non-Asian region, because their LBW was lower. Median predicted cycle 7 C_trough_ concentrations for patients ≤ 65 years was 82.7 μg/mL (range, 9.0–208.6 μg/mL) versus 80.8 μg/mL (32.7–156.9 μg/mL) for older patients (43 out of 489 were > 65 years). All (243/243) patients had trough levels above 20 μg/mL in the PH FDC SC arm and 99.6% (245/246) in the P + H IV arm.

**Fig. 3:**
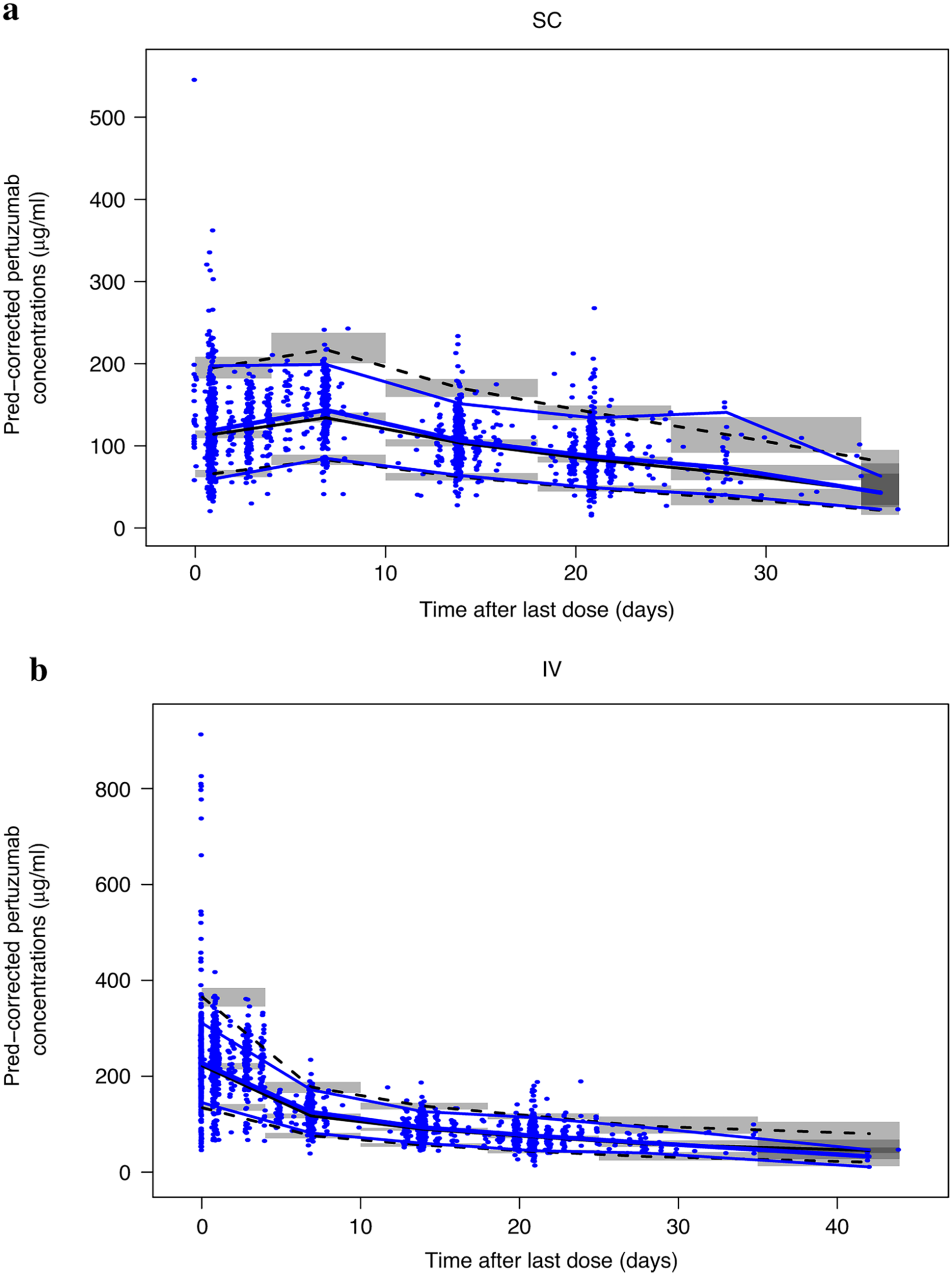
Prediction-corrected visual predictive check for pertuzumab after PH FDC SC (**A**) and P + H IV (**B**) administration. Blue dots represent observed pertuzumab concentrations. Blue lines represent the 5th, 50th, and 95th percentiles of observed pertuzumab concentrations. Black lines represent the 5th, 50th, and 95th percentiles of pertuzumab simulations. Gray bands represent the 95% prediction interval for corresponding black lines based on 500 simulations. *P* + *H IV* is intravenous pertuzumab and trastuzumab. *PH FDC SC* is fixed-dose combination of pertuzumab and trastuzumab for subcutaneous injection. Created using R version 3.6.0 (2019-04-26) in RStudio version 1.2.1335

Goodness-of-fit showed good agreement between predicted and observed concentrations, with no apparent bias in residuals over time and across predicted concentration values (Online Resource 2). The pcVPC for the model is shown in Fig. 3. The central tendency of the model and the 95th percentile ranges were comparable with the data for both the P + H IV and PH FDC SC cohorts.

### Pertuzumab-trastuzumab drug–drug interactions assessment

A total of 243 patients receiving PH FDC SC had 2092 evaluable trastuzumab samples. Overall, the central tendency and range of predicted trastuzumab concentrations by the HannaH model were comparable to those observed from the FeDeriCa data (Online Resource 3); the HannaH study collected trastuzumab samples at earlier time points compared with FeDeriCa and HannaH data appeared to have greater variability at the 95th percentile prediction range within the first 15 days after the dose.

NPC results based on 1000 simulations showed close agreement between the HannaH model and the FeDeriCa data: 1.9% of observations were above the 95th percentile, 48.8% above the 50th percentile, and 4.5% below the 5th percentile.

### Pertuzumab exposure–efficacy of PH FDC SC

**Fig. 4:**
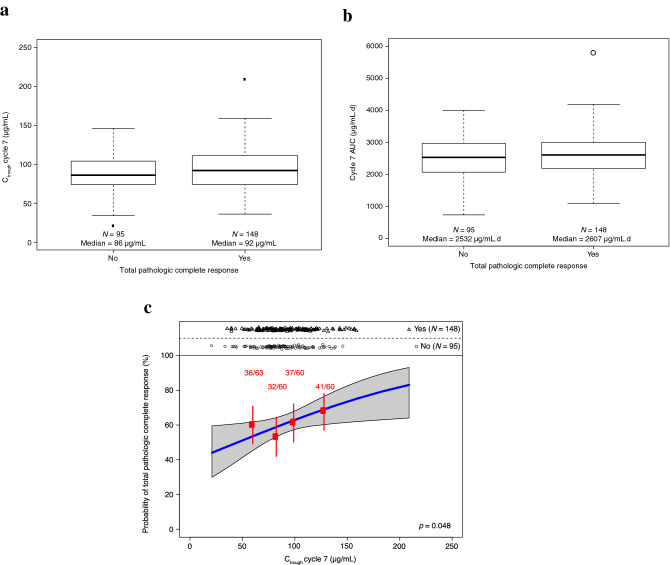
Model-predicted cycle 7 pertuzumab C_trough_ (**A**) and AUC (**B**) by tpCR, and univariate exposure–response relationship (**C**). Boxplots show the median (solid bold line), the interquartile range (shaded boxes), 1.5 times the interquartile range (whiskers), and points outside the whiskers (solid points). Univariate analysis red points indicate the mean concentration and response probability per exposure quartile. Vertical error bars indicate 90% confidence bounds. Numbers above each bar indicates patients with the event and the total number of patients in the quartile group. The blue line represents the fit of a logistic regression model. The shaded gray region indicates 90% confidence bands for the model fit. Points above the plot indicate model-predicted pertuzumab exposures in patients with a response versus exposures in patients with no response. *AUC* is area under the curve for a dosing interval (tau = 21). *C*_*max*_ is maximum concentration. *C*_*trough*_ is trough concentration. *tpCR* is total pathologic complete response. Created using R version 3.6.0 (2019-04-26) in RStudio version 1.2.1335

All 243 patients in the PH FDC SC arm with model-predicted pertuzumab cycle 7 C_trough_ were included in the analysis. Patients who achieved total pathologic complete response (tpCR; eradication of invasive disease in the breast and axilla, according to local pathologist assessment [ypT0/is, ypN0]) had a slightly higher median model-predicted pertuzumab cycle 7 C_trough_ (92 μg/mL) compared with those who did not (86 μg/mL) (Fig. 4A). Regardless of this difference in exposure, overall tpCR rates in the ER population were comparable: 60.9% in the PH FDC SC arm and 61.0% in the P + H IV arm. Median model-predicted AUC was similar in patients who achieved tpCR (2607 μg.d/mL versus 2532 μg.d/mL) (Fig. 4B).

There was no clear trend in tpCR across pertuzumab exposure quartiles (Table 2). Logistical regression for tpCR in the PH FDC SC arm indicated a slightly positive ER trend in the univariate case (*p* = 0.048) and after adjusting for baseline hormone receptor status (*p* = 0.069) (Fig. 4C). Differences in tpCR from the overall mean tpCR in the IV arm were small and ranged from − 7.6 to 7.4% in the second and third quartiles, respectively. After adjusting for baseline hormone receptor status, there was no statistically significant relationship between model-predicted pertuzumab exposures and tpCR rate (*p* = 0.069).

**Table 2:** Total pathologic complete response rate and safety parameters stratified by pertuzumab cycle 7 C_trough_ quartile in the PH FDC SC arm

	Pertuzumab cycle 7 C_trough_ quartile, µg/mL				
Endpoint	Q1 64 [21–74] (*n* = 63)	Q2 82 [75–89] (*n* = 60)	Q3 98 [90–109] (*n* = 60)	Q4 122 [110–209] (*n* = 60)	All 89 [21–209] (*n* = 243)
tpCR	38 (60.3%)	32 (53.3%)	37 (61.7%)	41 (68.3%)	148 (60.9%)
ΔtpCR	–0.7% (–14.2%, 12.9%)	–7.6% (–21.5%, 6.2%)	0.7% (–13.1%, 14.4%)	7.4% (–6.3%, 21.0%)	–

	Model-predicted pertuzumab Cycle 7 C_max_ quartile, µg/mL				
	Q1 [51–127] (*n* = 61)	Q2 [128–150] (*n* = 61)	Q3 [151–178] (*n* = 62)	Q4 [180–336] (*n* = 59)	All [51–336] (*n* = 243)
Grade ≥ 3 AE	13 (21.3%)	24 (39.3%)	14 (22.6%)	19 (32.2%)	70 (28.8%)
Serious AE	7 (11.5%)	9 (14.8%)	5 (8.1%)	4 (6.8%)	25 (10.3%)
Grade ≥ 3 diarrhea	2 (3.3%)	5 (8.2%)	3 (4.8%)	3 (5.1%)	13 (5.3%)
Grade ≥ 3 neutropenia	5 (8.2%)	13 (21.3%)	5 (8.1%)	10 (16.9%)	33 (13.6%)
Cardiac toxicity	7 (11.5%)	5 (8.2%)	7 (11.3%)	3 (5.1%)	22 (9.1%)
IRR (any grade) within 24 h, related to PH FDC SC	5 (8.2%)	6 (9.8%)	10 (16.1%)	3 (5.1%)	24 (9.9%)
Hypersensitivity/anaphylaxis (any grade), related to PH FDC SC	2 (3.3%)	0	0	0	2 (0.8%)

Numbers in square brackets indicate the range of pertuzumab cycle 7 C_trough/max_

*AE* is adverse event, *C*_*ma*x_ is maximum concentration, *C*_*trough*_ is trough concentration, *IRR* is injection-related reaction, *P* + *H IV* is intravenous pertuzumab and trastuzumab, *PH FDC SC* is fixed-dose combination of pertuzumab and trastuzumab for subcutaneous injection, *tpCR* is total pathologic complete response, *ΔtpCR* is difference from overall rate in IV arm (for comparison, in the P + H IV arm, rate of tpCR was 150/246 or 61.0%)

### Exposure–safety of PH FDC SC

There was a slightly higher rate of serious AEs in patients with lower exposures (1st and 2nd quartiles of model-predicted pertuzumab cycle 7 C_max_) compared with patients with higher exposures (3rd and 4th quartiles) (Table 2). Higher C_max_ quartiles were not associated with higher AE rates; accordingly, no trends with exposure were observed for grade ≥ 3 AEs, serious AEs, grade ≥ 3 diarrhea, grade ≥ 3 neutropenia, cardiac events, injection-related reactions of any grade within 24 h related to PH FDC SC, or any hypersensitivity/anaphylaxis related to PH FDC SC. Similar results were observed for cycle 7 AUC (data not shown).

## Discussion

Pertuzumab popPK following PH FDC SC or P + H IV administration in FeDeriCa was described adequately by a two-compartment model with first-order absorption. Statistically significant covariates on pertuzumab exposure (cycle 7 C_trough_) included LBW, race, and albumin; however, their effects were relatively small compared with the overall inter-individual variability of the population and the effects were not considered clinically relevant. Thus, no dose adjustments of PH FDC SC based on these covariates are warranted. As described above, cycle 7 was selected for pertuzumab exposure metrics, as this was when the majority of patients reached steady state. When investigating lean LBW, heavier patients had a lower exposure, and lighter patients a higher exposure, due to fixed dosing, as expected. The interquartile range of exposures, as well as the whiskers of the plots, were all greatly overlapping across LBW quartiles. There was no apparent relationship suggestive of an increase in severe or serious AE rates with increasing exposure or decreasing body weight for H SC [29]. Overall, similar to the experience with P + H IV/SC, there did not appear to be an impact of LBW on safety of PH FDC SC; however, the low numbers of patients/events in the FeDeriCa subgroups preclude definitive conclusions. Additionally, even in heavier patients (LBW > 48.8 kg), all patients following PH FDC SC administration achieved the target efficacious steady-state trough concentration of 20 µg/mL at pre-dose cycle 8.

Furthermore, similar to the previous P + H IV analysis [31, 32], PH FDC SC administration did not show a clinically meaningful ER relationship between tpCR and AE rates, and was found to demonstrate a manageable safety profile and a comparable efficacy to P + H IV.

The FeDeriCa P population PK model is a two-compartment model with first-order SC absorption with estimated bioavailability of 71%. The bioavailability estimated here was consistent with the findings from the phase Ib SC pertuzumab dose-finding study [28]. The high shrinkage for F that was observed was expected, as only half of the population had IV data and none of the patients received both the IV and SC formulations.

The model demonstrates a smaller clearance (0.163 L/d for current model; 0.235 L/d for previous model) and similar volume estimates than previous estimates for P + H IV, based on a dataset with a slightly smaller median LBW and larger median albumin [27]. The estimated pertuzumab clearance for a typical patient in the FeDeriCa study using the previous P IV model [27] is 0.204 L/d.

Similar to previous studies of PK of P + H IV administration, baseline serum albumin and LBW were found not to be clinically relevant for pertuzumab exposure for PH FDC SC, indicating that variations in these covariates do not require dose adjustments. In the current study, the Asian region was associated with 12% increase in CL; in contrast to the 100 Asian patients included here, the previous IV pertuzumab popPK analysis in 2014 had only 22 (all of which were Japanese), and did not appear to impact on CL in a similar way. Nevertheless, in the present study, a similar CL and exposure in Asian patients compared to the overall population is expected when accounting for the lower LBW of this subgroup.

Trastuzumab concentrations in the PH FDC SC arm were described adequately by the previously developed trastuzumab popPK model from HannaH data [29]. The NPC showed that ~ 50% of observed trastuzumab concentrations in the PH FDC SC arm of the FeDeriCa study were above and below the 50th percentile prediction from the HannaH population model [29], suggesting that pertuzumab does not affect trastuzumab PK as part of the PH FDC SC. This was consistent with IV data for the combination [27, 31, 32] and may be due to the fact that HER2 epitope binding between the two antibodies is different with no steric hindrance (meaning that target-mediated drug–drug interactions were unlikely) [1]. It may also be due to the fact that target-mediated drug–drug interactions are saturated at the current dose of PH FDC SC and both monoclonal antibodies are eliminated through large-capacity, non-specific, Fc receptor-mediated immunoglobulin G clearance mechanisms [33].

Differences in tpCR rate were not associated with differences in median model-predicted pertuzumab cycle 7 C_trough_, and the response across all exposure quartiles was similar to that in the P + H IV arm. In a similar analysis of the NeoSphere study, which utilized P + H IV, there was no association between the pertuzumab serum concentration and pCR in patients with HER2-positive breast cancer [31]. In the present study, lower rates of tpCR were observed in patients with hormone receptor-positive status, but this trend was not statistically significant. Therefore, the detected numerical difference should be interpreted with caution; this observation may be attributed to: (1) the ten-fold range of P exposures (21–209 µg/mL) in the PH FDC SC arm; (2) the relatively large sample size (*n* = 243) compared with previous IV-based studies; or (3) may be an artifact caused by a confounding disease status factor that could influence both exposure and response, which have been observed for other biologics for oncology indications (although this is not normally the case in the early breast cancer setting) [32, 34].

All patients in the PH FDC SC arm achieved model-predicted C_trough_ exceeding 20 μg/mL, which is the targeted effective concentration identified from preclinical mouse xenograft models and early clinical response data [27]. In the present study, higher model-predicted pertuzumab cycle 7 C_max_ and AUC were not associated with increased probability of grade ≥ 3 AEs or increased probability of cardiac toxicity, serious AEs, grade ≥ 3 diarrhea, grade ≥ 3 neutropenia, injection-related reactions (any grade) within 24 h related to PH FDC SC, nor was hypersensitivity/anaphylaxis (any grade) related to PH FDC SC.

The ER of SC trastuzumab was well characterized in the HannaH study [29]. In HannaH, fixed 600 mg SC dose of trastuzumab provided the desired exposure, with steady-state C_trough_ (35–123 µg/mL for the 5th–95th percentiles) above the historical target concentration of 20 µg/mL for efficacy. Fixed dosing was further supported by lack of an ER relationship between PK, tpCR, and grade ≥ 3 AEs, and no dose adjustments were required for covariates such as albumin or LBW.

Limitations of the current analysis include a limited number of AEs (therefore, results should be interpreted cautiously) and the fact that only one dose level was observed and studied, which limits our ability to describe the exposure–efficacy and exposure–safety relationships. In addition, while shrinkage for CL and *V*_*c*_ was low, shrinkage for *F* and *V*_*p*_ were higher, which may have impacted the model-predicted exposures.

Overall, available popPK and ER analyses for the FeDeriCa study, in conjunction with clinical experience with IV pertuzumab, IV trastuzumab, and SC trastuzumab, indicates that the current dose of PH FDC SC (loading dose: 1200 mg pertuzumab and 600 mg trastuzumab in 15 mL; maintenance dose: 600 mg pertuzumab and trastuzumab in 10 mL, and 2000 U/mL recombinant human hyaluronidase) provides a positive benefit–risk profile with comparable efficacy with P + H IV that is achieved with a manageable safety profile. In addition, PH FDC SC provides healthcare professionals with the possibility of administering treatment in the patient’s home, as noted by the FDA [15]; an option that has become more important recently due to the COVID-19 pandemic. Home administration potentially reduces the risk of patients acquiring COVID-19 infection during hospital visits, a risk that could in turn lead to increased complications in patients with cancer [16]. The results further suggest that, in practice, no dose modifications are required according to covariates such as albumin, LBW, and race.

## Data-sharing statement

Qualified researchers may request access to individual patient-level data through the clinical study data request platform: https://vivli.org/. Further details on Roche's criteria for eligible studies are available here: https://vivli.org/members/ourmembers. For further details on Roche’s Global Policy on the Sharing of Clinical Information and how to request access to related clinical study documents, see here: https://www.roche.com/research_and_development/who_we_are_how_we_work/clinical_trials/our_commitment_to_data_sharing.htm.

## Supplementary Information

Below is the link to the electronic supplementary material.

Supplementary file1 (DOCX 663 KB)
