# Supplementary material for: Population pharmacokinetic and exploratory exposure–response analysis of the fixed-dose combination of pertuzumab and trastuzumab for subcutaneous injection in patients with HER2-positive early breast cancer in the FeDeriCa study
Source: Cancer Chemother Pharmacol. 2021 Jun 9;88(3):499–512. doi: 10.1007/s00280-021-04296-0 (PMC8187458; doi:10.1007/s00280-021-04296-0)

**Electronic supplementary material**

Population pharmacokinetic and exploratory exposure–response analysis of the fixed-dose combination of pertuzumab and trastuzumab for subcutaneous injection in patients with HER2-positive early breast cancer in the FeDeriCa study

Bei Wang^1^ (0000-0002-9530-0385) · Rong Deng^1^ (0000-0003-2005-7517) ·
Stefanie Hennig^2^ (0000-0001-5972-3711) · Tanja Badovinac Crnjevic^3^
(0000-0002-0922-3409) · Monika Kaewphluk^3^ (0000-0002-0637-7509) ·
Matts Kågedal^1^ (0000-0002-1261-0354) · Angelica L. Quartino^1^* (0000-0003-0184-4670) · Sandhya Girish^1^^†^ (0000-0002-9336-1600) · Chunze Li^1^ (0000-0002-8906-6553) ·
Whitney P. Kirschbrown^1^ (0000-0003-0580-5425)

^1^Genentech, Inc., South San Francisco, CA, USA

^2^Certara, Inc., Princeton, NJ, USA

^3^F. Hoffmann-La Roche Ltd, Basel, Switzerland

*Current Affiliation: Clinical Pharmacology and Quantitative Pharmacology, AstraZeneca, Gothenburg, Sweden

^†^Current Affiliation: Gilead Sciences, Inc., Foster City, CA, USA

**Corresponding author:**

Dr. Whitney P. Kirschbrown, PharmD, PhD

Genentech, Inc., 1 DNA Way, South San Francisco, CA 94080, USA.

Phone: 650-467-3808

Email: kirschbrown.whitney@gene.com

ORCID ID: 0000-0003-0580-5425

**Online Resource 1** Baseline categorical and continual covariates

| **Covariate** | **P + H IV** | **PH FDC SC** | **All patients** |
| --- | --- | --- | --- |
| Route of administration |  |  |  |
| SC | 0 | 243 (100%) | 243 (49.7%) |
| IV | 246 (100%) | 0 | 246 (50.3%) |
| Sex |  |  |  |
| Male | 2 (0.8%) | 0 | 2 (0.4%) |
| Female | 244 (99.2%) | (243 (100%) | 487 (99.6%) |
| Ethnic origin |  |  |  |
| White | 161 (65.4%) | 163 (67.1%) | 324 (66.3%) |
| Asian | 51 (20.7%) | 50 (20.6%) | 101 (20.7%) |
| Black or African American | 3 (1.2%) | 3 (1.2%) | 6 (1.2%) |
| Unknown/other | 31 (12.6%) | 27 (11.1%) | 58 (11.9%) |
| Ethnicity |  |  |  |
| Hispanic | 32 (13.0%) | 41 (16.9%) | 73 (14.9%) |
| Other | 214 (87.0%) | 202 (83.1%) | 416 (85.1%) |
| Study arm |  |  |  |
| P + H IV | 246 (100) | 0 | 246 (50.3%) |
| PH FDC SC | 0 | 243 (100%) | 243 (49.7%) |
| Type of chemotherapy |  |  |  |
| ddAC followed by paclitaxel | 118 (48.0%) | 118 (48.6%) | 236 (48.3%) |
| AC followed by docetaxel | 128 (52.0%) | 125 (51.4%) | 253 (51.7%) |
| Hormone receptor status |  |  |  |
| Estrogen and progesterone  receptor-negative | 103 (41.9%) | 90 (37.0%) | 193 (39.5%) |
| Estrogen or progesterone  receptor-positive | 142 (57.7%) | 151 (62.1%) | 293 (59.9%) |
| Estrogen or progesterone  receptor status unknown | 1 (0.4%) | 2 (0.8%) | 3 (0.6%) |
| Clinical stage at presentation |  |  |  |
| II–IIIA | 198 (80.5%) | 192 (79.0%) | 390 (79.8%) |
| IIIB–IIIC | 48 (19.5%) | 51 (21.0%) | 99 (20.2%) |
| ECOG performance status |  |  |  |
| 0 | 225 (91.5%) | 220 (90.5%) | 445 (91.0%) |
| 1 | 15 (6.1%) | 20 (8.2%) | 35 (7.2%) |
| Unknown | 6 (2.4%) | 3 (1.2%) | 9 (1.8%) |
| Asian region |  |  |  |
| No | 196 (79.7%) | 193 (79.4%) | 389 (79.6%) |
| Yes | 50 (20.3%) | 50 (20.6%) | 100 (20.4%) |
| Age |  |  |  |
| ≤ 65 years | 222 (90.2%) | 224 (92.2%) | 446 (91.2%) |
| > 65 years | 24 (9.7%) | 19 (7.8%) | 43 (8.8%) |
| Renal function |  |  |  |
| Normal | 153 (62.2%) | 152 (62.6%) | 305 (62.4%) |
| Mild impairment | 81 (32.9%) | 77 (31.7%) | 158 (32.3%) |
| Moderate impairment | 12 (4.9%) | 14 (5.8%) | 26 (5.3%) |
| Hepatic function |  |  |  |
| Normal | 226 (91.9%) | 222 (91.4%) | 448 (91.6%) |
| Mild impairment | 18 (7.3%) | 20 (8.2%) | 38 (7.8%) |
| Moderate impairment | 2 (0.8%) | 1 (0.4%) | 3 (0.6%) |
| **Demographic** |  |  |  |
| Lean body weight, kg | 45.4 (33.9, 73.0) | 44.9 (29.8, 60.7) | 45.1 (29.8, 73.0) |
| Serum albumin, g/L | 43.0 (35.8, 52.0) | 43.8 (35.0, 57.0) | 43.2 (35.0, 57.0) |
| Weight, kg | 65.1 (41.0, 126.0) | 64.6 (39.2, 136.0) | 65.0 (39.2, 136.0) |
| Height, cm | 162 (145.0, 185.0) | 161 (146.0, 179.0) | 162 (145.0, 185.0) |
| Body surface area, m^2^ | 1.7 (1.3, 2.4) | 1.7 (1.3, 2.4) | 1.7 (1.3, 2.4) |
| BMI, kg/m^2^ | 25.1 (16.4, 43.2) | 25 (16.7, 55.5) | 25.1 (16.4, 55.5) |
| Age, years | 49.0 (27.0, 75.0) | 51.0 (25.0, 80.0) | 51.0 (25.0, 80.0) |
| Aspartate transaminase, U/L | 19.0 (5.0, 57.0) | 19.0 (9.0, 94.0) | 19.0 (5.0, 94.0) |
| Total bilirubin, mg/dL | 8.6 (1.7, 28.7) | 8.6 (1.2, 32.3) | 8.6 (1.2, 32.3) |
| Alanine transaminase, U/L | 18.0 (6.0, 86.0) | 17.0 (4.1, 115.0) | 17.6 (4.1, 115.0) |
| Serum creatinine, μmol/L | 63.3 (29.2, 108.0) | 61.9 (37.1, 106.0) | 62.0 (29.2, 108.0) |
| Creatinine clearance, mL/min | 96.4 (46.3, 202.0) | 96.4 (42.2, 228.0) | 96.4 (42.2, 228.0) |

Categorial values are number of patients (%) and continuous values are median (range)

*BMI* is body mass index. *(dd)AC* is (dose-dense) doxorubicin + cyclophosphamide. *ECOG* is Eastern Cooperative Oncology Group. *IV* is intravenous. *P + H IV* is intravenous pertuzumab and trastuzumab. *PH FDC SC* is fixed-dose combination of pertuzumab and trastuzumab for subcutaneous injection. *SC* is subcutaneous

**Online Resource 2** Final model goodness-of-fit plots in the PH FDC SC (A) and P + H IV (B) arms. Top panels: Observed versus population and individual predicted concentrations (ln-scale). Middle panels: CWRES versus population prediction (log-scale) and time. Bottom panels: absolute values of individual WRES versus individual predictions (log-scale) for the final PK model. *CWRES* is population conditional weighted residuals. *P + H IV* is intravenous pertuzumab and trastuzumab. *PH FDC SC* is fixed-dose combination of pertuzumab and trastuzumab for subcutaneous injection. *WRES* is weighted residuals

Created using R version 3.6.0 (2019-04-26) in RStudio version 1.2.1335

**(a)**


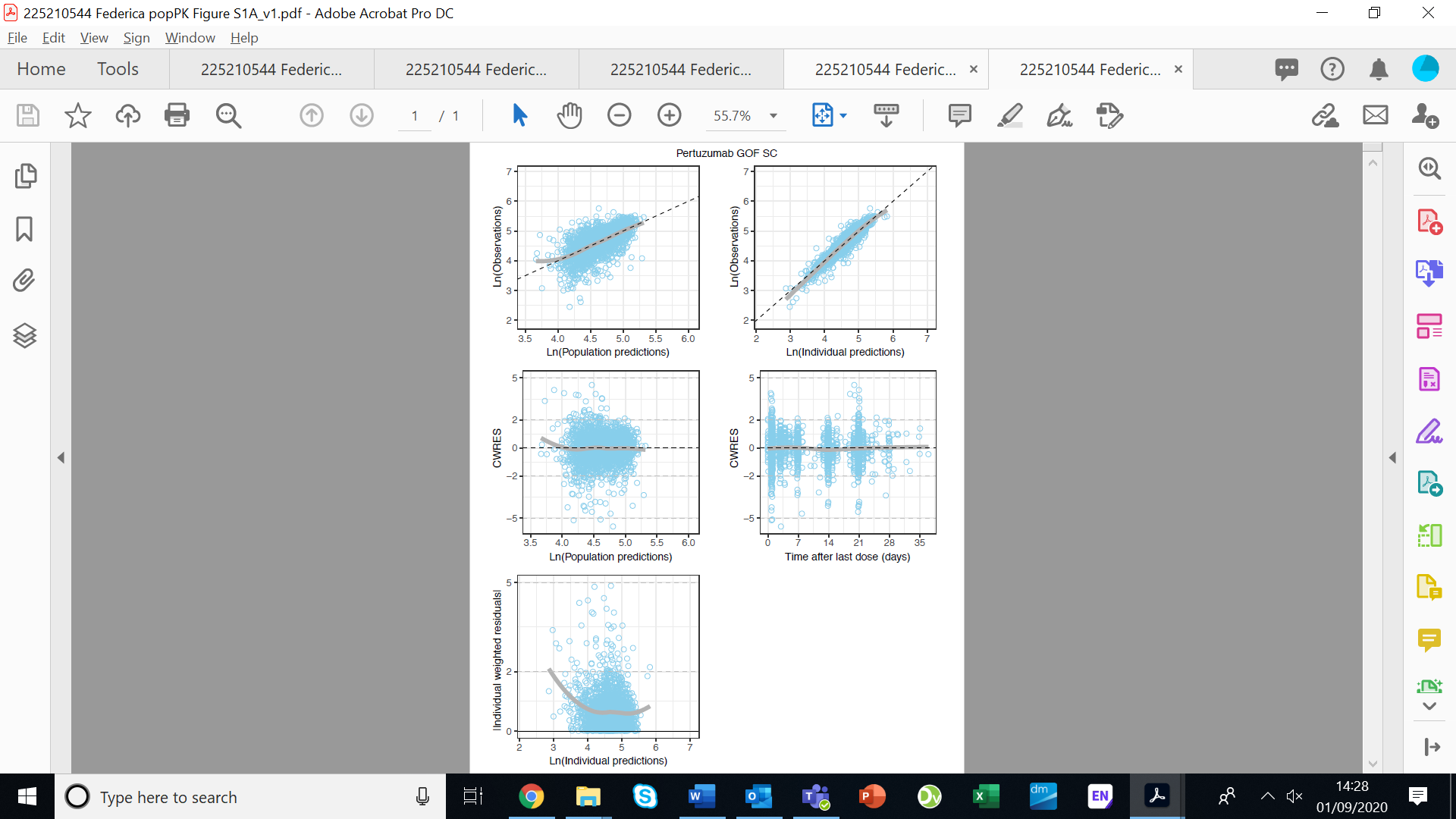


**(b)**


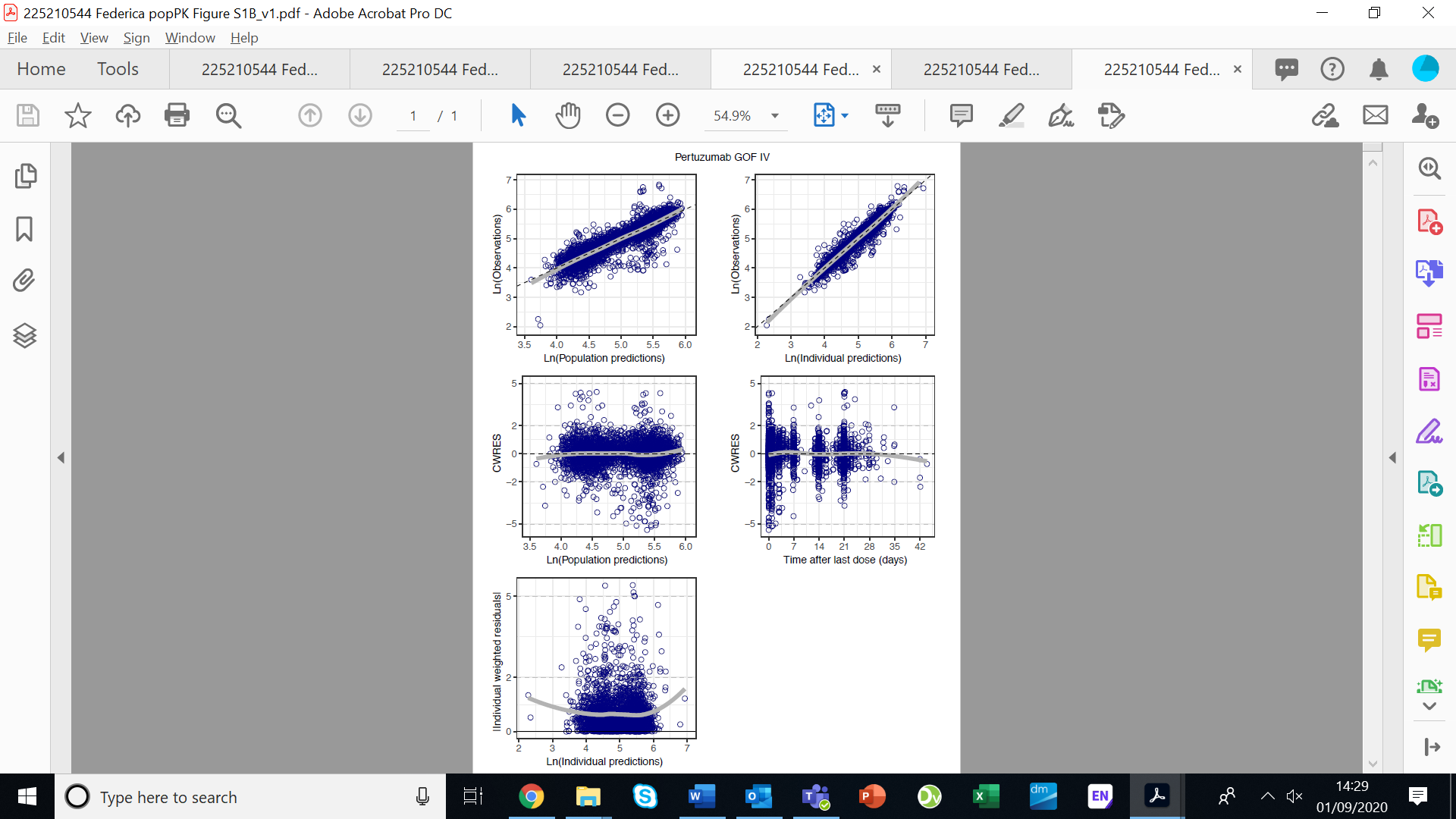


**Online Resource 3** Prediction-corrected visual predictive check for trastuzumab concentration using the HannaH trastuzumab population pharmacokinetic model. Blue dots represent observed trastuzumab concentrations in the PH FDC SC arm. Blue lines represent the 5^th^, 50^th^, and 95^th^ percentiles of observed trastuzumab concentrations in the PH FDC SC arm. Black lines represent the 5^th^, 50^th^, and 95^th^ percentiles of trastuzumab simulations using the HannaH trastuzumab population pharmacokinetic model. Grey bands represent 95% prediction interval for corresponding black lines based on 1000 simulations. *PH FDC SC* is fixed-dose combination of pertuzumab and trastuzumab for subcutaneous injection

Created using R version 3.6.0 (2019-04-26) in RStudio version 1.2.1335


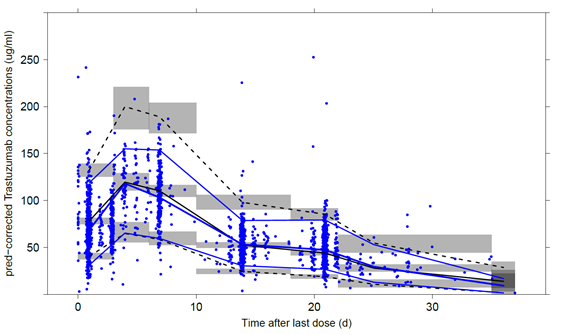

Supplement: Supplementary file 1 — Supplementary file1 (DOCX 663 KB) [file 280_2021_4296_MOESM1_ESM.docx]
